# Supplementary material for: Epithelial-to-Mesenchymal Transition Enhances Cancer Cell Sensitivity to Cytotoxic Effects of Cold Atmospheric Plasmas in Breast and Bladder Cancer Systems
Source: Cancers (Basel). 2021 Jun 9;13(12):2889. doi: 10.3390/cancers13122889 (PMC8226878; doi:10.3390/cancers13122889)
Supplement: Supplementary file 1 [file cancers-13-02889-s001.zip › cancers-1196209-supplementary.pdf]

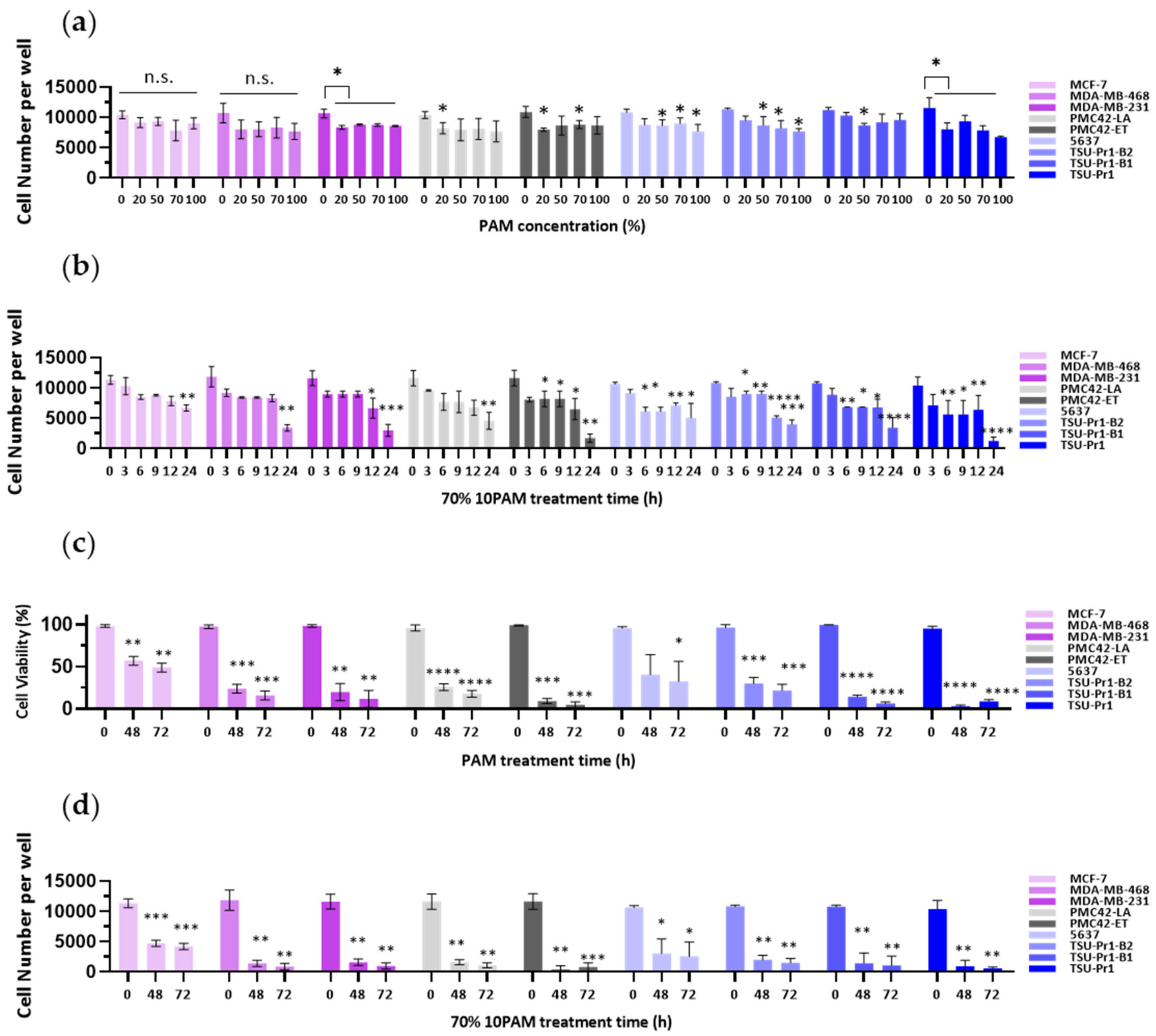

Supplementary Figure S1. Effects of PAM on cell numbers. Breast cancer cell lines (MDA-MB-231/MDA-MB-468/MCF-7; pink) and EMT/MET cell line systems in breast (PMC42-ET/LA; grey) and bladder (5673/TSU-Pr1/B1/B2; blue) cancer were starved with serum free medium for 3h and treated with PAM in 0%, 20%, 50%, 70% and 100% 10PAM for 12 hours (**a**) or 70% 10PAM for 0h, 3h, 6h, 9h, 12h and 24h (**b**). Cell number from the Hoechst 33342 results from Live Dead Cell Assay (mean -/+ SEM of 0% or 0h PAM, respectively) was calculated by In Carta analysis; the dose response for 12h treatment is shown (a), as are the cell viability (**c**) and cell number (**d**) after 70% PAM treatment for 48h and 72h. The mean and SEM of the average for each data point from each of the 3 experiments are shown. "*" represents a $p<0.05$, "**" represents a $p<0.01$, "***" represents a $p<0.001$ and "****" represents a $p<0.0001$.

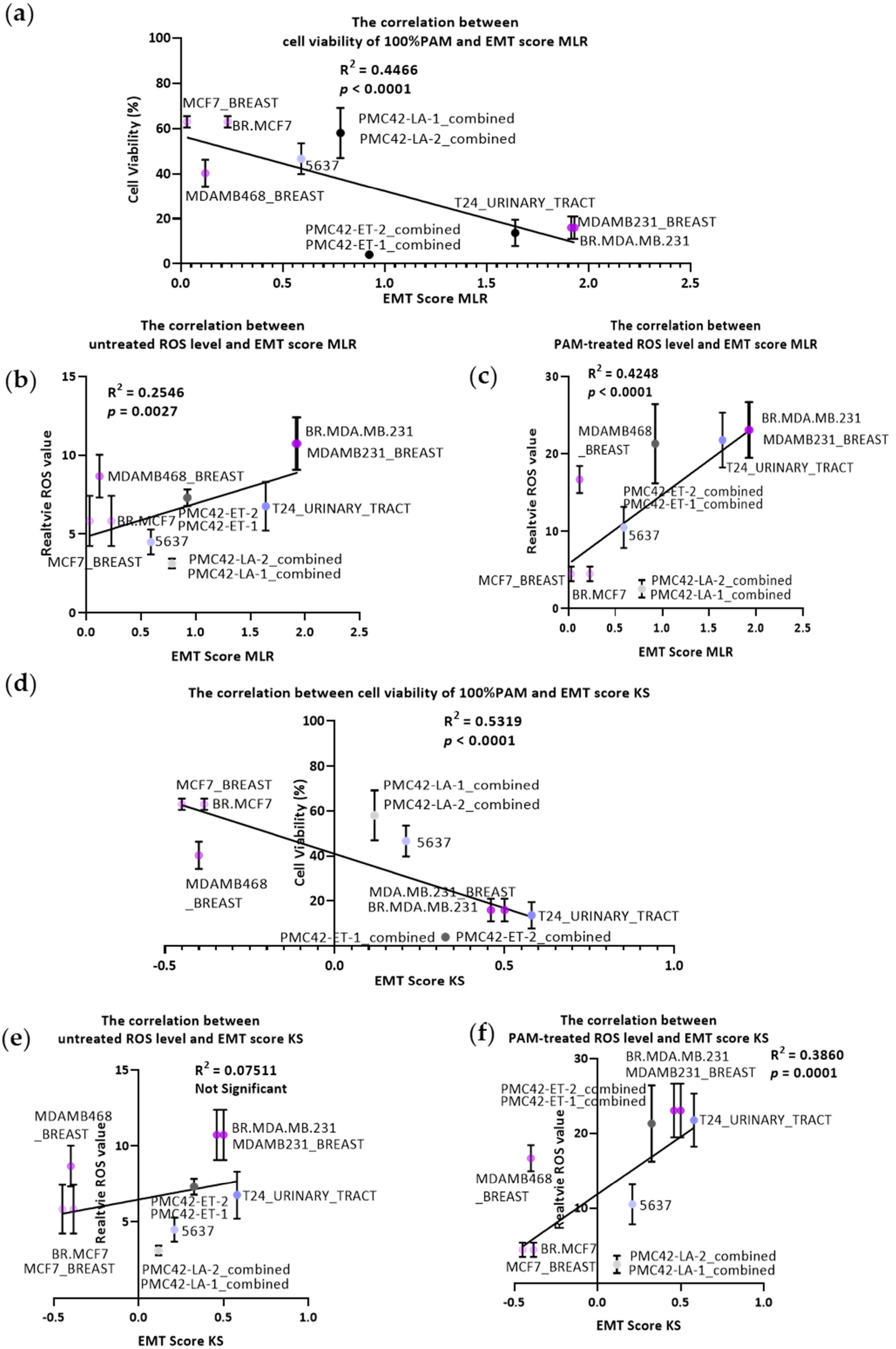
(a)
The correlation between
cell viability of 100%PAM and EMT score MLR
R2 = 0.4466
p < 0.0001
Cell Viability (%)
EMT Score MLR
MCF7_BREAST
BR.MCF7
PMC42-LA-1_combined
PMC42-LA-2_combined
5637
MDAMB468_BREAST
T24_URINARY_TRACT
MDAMB231_BREAST
BR.MDA.MB.231
PMC42-ET-2_combined
PMC42-ET-1_combined
(b)
The correlation between
untreated ROS level and EMT score MLR
R2 = 0.2546
p = 0.0027
Realtvie ROS value
EMT Score MLR
BR.MDA.MB.231
MDAMB231_BREAST
MDAMB468_BREAST
BR.MCF7
PMC42-ET-2
PMC42-ET-1
T24_URINARY_TRACT
5637
MCF7_BREAST
PMC42-LA-2_combined
PMC42-LA-1_combined
(c)
The correlation between
PAM-treated ROS level and EMT score MLR
R2 = 0.4248
p < 0.0001
Realtvie ROS value
EMT Score MLR
MDAMB468
_BREAST
BR.MDA.MB.231
MDAMB231_BREAST
T24_URINARY_TRACT
PMC42-ET-2_combined
PMC42-ET-1_combined
5637
MCF7_BREAST
BR.MCF7
PMC42-LA-2_combined
PMC42-LA-1_combined
(d)
The correlation between cell viability of 100%PAM and EMT score KS
R2 = 0.5319
p < 0.0001
Cell Viability (%)
EMT Score KS
MCF7_BREAST
BR.MCF7
PMC42-LA-1_combined
PMC42-LA-2_combined
5637
MDAMB468
_BREAST
MDA.MB.231_BREAST
BR.MDA.MB.231
T24_URINARY_TRACT
PMC42-ET-1_combined
PMC42-ET-2_combined
(e)
The correlation between
untreated ROS level and EMT score KS
R2 = 0.07511
Not Significant
Realtvie ROS value
EMT Score KS
MDAMB468
_BREAST
BR.MDA.MB.231
MDAMB231_BREAST
PMC42-ET-2
PMC42-ET-1
T24_URINARY_TRACT
5637
BR.MCF7
MCF7_BREAST
PMC42-LA-2_combined
PMC42-LA-1_combined
(f)
The correlation between
PAM-treated ROS level and EMT score KS
R2 = 0.3860
p = 0.0001
Realtvie ROS value
EMT Score KS
BR.MDA.MB.231
MDAMB231_BREAST
PMC42-ET-2_combined
PMC42-ET-1_combined
T24_URINARY_TRACT
MDAMB468
_BREAST
5637
BR.MCF7
MCF7_BREAST
PMC42-LA-2_combined
PMC42-LA-1_combined

Supplementary Figure S2. MLR-derived EMT scores correlate with PAM response. (**a**) The correlation between EMT score calculated by MLR and cell viability of MCF-7, MDA-MB-468, MDA-MB-231, PMC42-LA, PMC42-ET, 5637 and TSU-Pr1(T24) cells after 12h treatment with 100% PAM. (**b**) The correlation between EMT score (MLR) and untreated ROS of the same cell lines as shown in (a). (**c**) The correlation between EMT score (MLR) and 12h 70% PAM-treated ROS level of the same cell line as shown in (a). KS-derived EMT scores correlate with PAM response. (**d**) The correlation between EMT score calculated by KS and cell viability of MCF-7, MDA-MB-468, MDA-MB-231, PMC42-LA, PMC42-ET, 5637 and TSU-Pr1(T24) cells after 12h treatment with 100% PAM. (**e**) The correlation between EMT score (KS) and untreated ROS of the same cell lines as shown in (a). (**f**) The correlation between EMT score (KS) and 12h 70% PAM-treated ROS levels in the same cell lines as shown in (a).

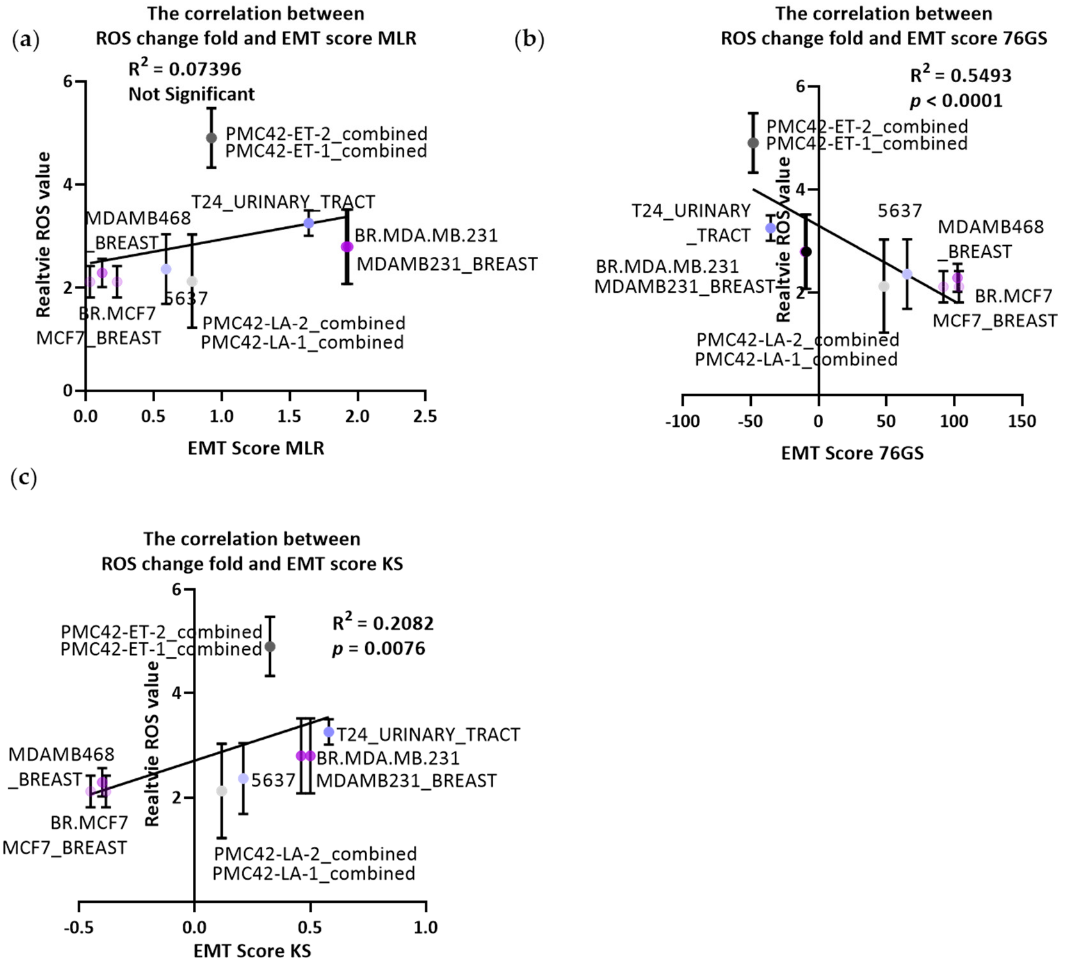

Supplementary Figure S3. The correlation between EMT score and ROS fold change after PAM treatment. (**a**) The correlation between EMT score calculated by MLR and ROS fold change of MCF-7, MDA-MB-468, MDA-MB-231, PMC42-LA, PMC42-ET, 5637 and TSU-Pr1(T24) cell lines. (**b**) The correlation between EMT score (76GS) and ROS fold change of the same cell lines as shown in (a). (**c**) The correlation between EMT score (KS) and ROS fold change of the same cell lines as shown in (a).

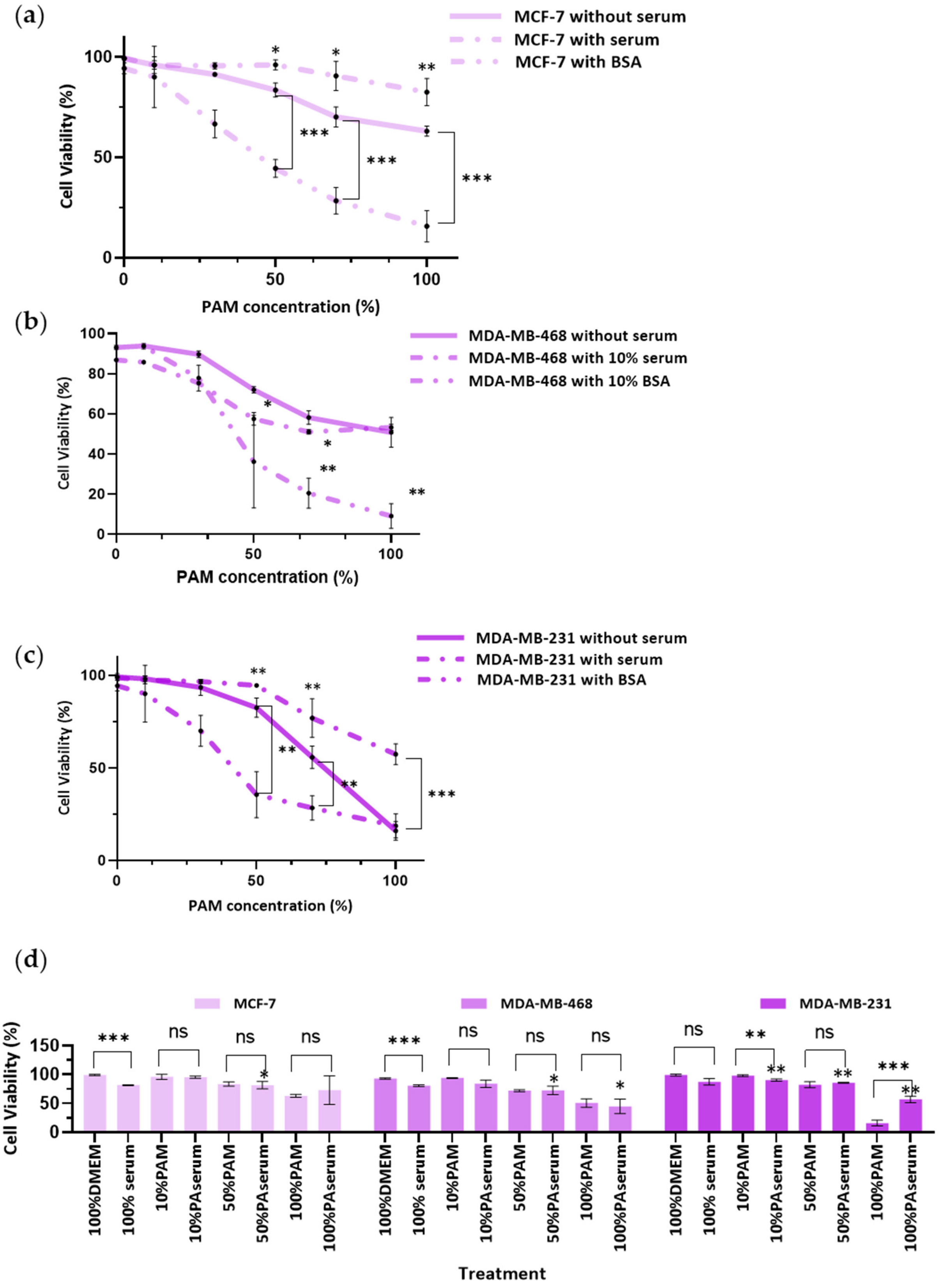

Supplementary Figure S4. MCF-7 (**a**), MDA-MB-468 (**b**), MDA-MB-231(**c**) were treated with 0%, 25%, 50%, 75% and 100% PAM for 12h in the absence or presence of FBS (10%) or BSA (10%) and cell viability was calculated by live-dead cell assay. (**d**) The MCF-7, MDA-MB-468 and MDA-MB-231 cells were treated with 0% (100% DMEM), 10% PAM, 100% PAM or PAserum with DMEM dilution for 12h. The

cell viability was tested as per (a). Statistical comparisons are shown between PAM and PASerum at each dose (above the line), and also comparing PASerum at each concentration to 100% DMEM (above the bars). The mean and SEM of the average for each data point from each of the 3 experiments are shown. "*" represents a $p<0.05$, "**" represents a $p<0.01$, and "***" represents a $p<0.001$.

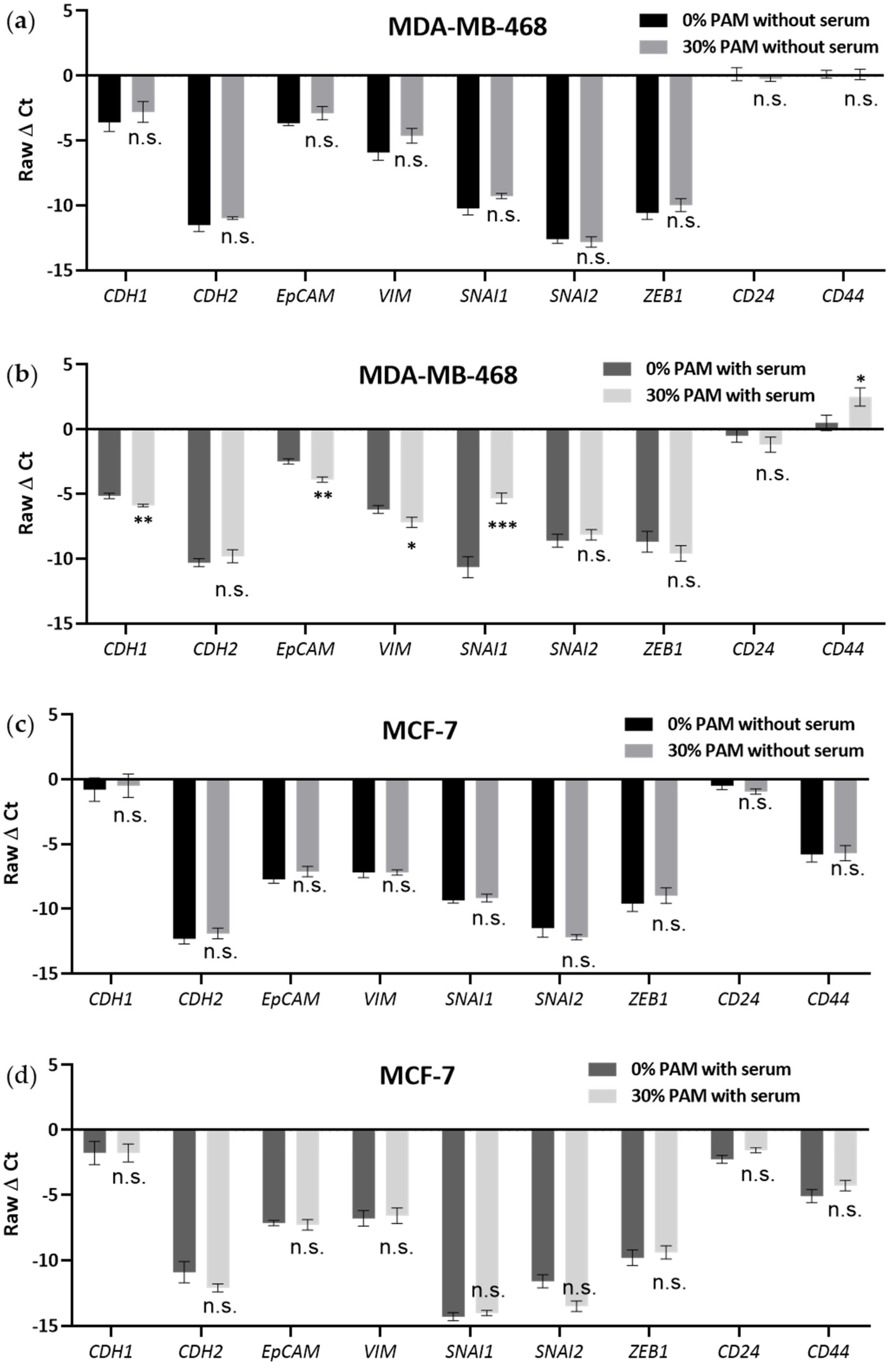
(a)
MDA-MB-468
0% PAM without serum
30% PAM without serum
Raw Δ Ct
5
0
-5
-10
-15
CDH1
CDH2
EpCAM
VIM
SNAI1
SNAI2
ZEB1
CD24
CD44
n.s.
(b)
MDA-MB-468
0% PAM with serum
30% PAM with serum
**
*
***
n.s.
(c)
MCF-7
0% PAM without serum
30% PAM without serum
(d)
MCF-7
0% PAM with serum
30% PAM with serum

Supplementary Figure S5. EMT marker changes after PAM treatment without or with serum. MDA-MB-468 cells were plated overnight in 48-well-plates and treated with 0% or 30% PAM without (a) or with 10% serum (b) for 3 days. Three entirely independent biological replicates were assessed. MCF-7 cells were plated as per (a) and treated with 0% or 30% PAM without (c) or with 10% serum (d) for 3 days. Epithelial marker (CHD1), mesenchymal markers (CDH2, EpCAM, VIM, SNAI1, SNAI2, ZEB1) and stemness markers (CD24, CD44) were detected via real-time RTqPCR. The mean and SEM of the average for each data point from each of the 3 experiments are shown. "*" represents $p<0.05$, "**" represents $p<0.01$, and "***" represents $p<0.001$.
